# Tramadol use is associated with reduced 28-day mortality in ICU patients after cardiac surgery: a retrospective study based on the MIMIC-IV database

**DOI:** 10.3389/fphar.2026.1770570

**Published:** 2026-01-16

**Authors:** Jingyan Xu, Yang Zhang, Rongqing Gao

**Affiliations:** Department of Anesthesiology, The Second Affiliated Hospital Zhejiang University School of Medicine, Hangzhou, Zhejiang, China

**Keywords:** 28-day mortality, cardiac surgery, intensive care unit, MIMIC-IV, tramadol

## Abstract

**Background:**

To evaluate the association between tramadol use and short-term outcomes, including 28-day mortality, intensive care unit (ICU) mortality, and ICU length of stay, in critically ill patients undergoing cardiac surgery.

**Methods:**

This retrospective cohort study included 3,544 participants from the MIMIC-IV database. A comprehensive analytical approach was employed, including multivariate Cox regression, subgroup analysis, propensity score matching (PSM), inverse probability weighting (IPW), doubly robust estimation, and E-value calculation. Receiver operating characteristic (ROC) curves and the DeLong test were used to compare the predictive performance of different opioids, and SHAP analysis was employed for model interpretation.

**Results:**

Tramadol use was consistently associated with a significant reduction in 28-day mortality across all models. The hazard ratios (HR) ranged from 0.305 to 0.341 after rigorous adjustment and matching (all *P* < 0.05). Subgroup analyses demonstrated the robustness of this protective association, and a significant interaction was observed with respect to surgery type after PSM. Furthermore, tramadol demonstrated superior predictive performance for 28-day mortality (AUC = 0.603) compared to other opioids, including fentanyl, hydromorphone, morphine, and oxycodone AUC range: 0.523–0.597). However, no significant association was found with secondary outcomes like ICU mortality or length of stay.

**Conclusion:**

Tramadol administration is independently associated with a significantly lower risk of 28-day mortality in cardiac surgery patients, showing better predictive utility than other common opioids, which may inform postoperative analgesic strategies.

## 1 Introduction

Cardiac surgery is a critical intervention for severe cardiovascular diseases. It serves as a cornerstone therapy for conditions ranging from coronary artery disease to valvular heart disease (Ruel and Chikwe, 2024; Unger et al., 2018). With an aging population and increasing prevalence of cardiovascular disorders, the volume of cardiac surgeries continues to rise, with approximately 2 million procedures performed worldwide annually (Shamsi et al., 2025). Substantial advances in surgical techniques and perioperative care have significantly improved patient survival (Pei et al., 2025). Nevertheless, postoperative mortality remains a major concern, influenced by comorbidities, surgical complexity, and management strategies. U.S. national data report an in-hospital mortality rate of approximately 2.1% among cardiac surgery patients (Devereaux et al., 2022), underscoring the ongoing need for enhanced risk stratification and individualized treatment approaches to optimize patient prognosis.

Pain management is a vital component of perioperative care in cardiac surgery, with opioid-based analgesia forming the therapeutic foundation (Zhong et al., 2024). However, selecting the optimal analgesic regimen requires careful balancing of efficacy against potential adverse effects. Tramadol, a centrally acting analgesic, plays a unique role in this balance. It functions as a weak μ-opioid receptor agonist while simultaneously inhibiting serotonin and norepinephrine reuptake, conferring a dual mechanism of action that differentiates it from traditional opioids (Beakley et al., 2015; Bravo et al., 2017). This pharmacological profile supports its role as a strategic “step-down” analgesic in the ICU, enabling a smooth transition from potent intravenous agents to oral therapy and serving as a core component of multimodal analgesia designed to preserve respiratory drive and hemodynamic stability (Miotto et al., 2017; Mo et al., 2020).

Despite these theoretical advantages, the comparative efficacy of tramadol relative to other standard opioids used in cardiac surgery—such as fentanyl, morphine, and hydromorphone—remains insufficiently studied. In current practice, potent opioids like fentanyl are favored for hemodynamic stability but are associated with prolonged mechanical ventilation and postoperative ileus (Okano et al., 2025; Burkhard et al., 2023). Conversely, morphine provides effective analgesia but may trigger histamine release and vasodilation (Parodi et al., 2023). Tramadol’s potential clinical value lies in its capacity to offer a “middle ground,” delivering adequate analgesia during the recovery phase while potentially preserving organ function and modulating immune responses more favorably (Bradley and Boland, 2023). However, whether these pharmacological distinctions translate into measurable survival benefits in high-risk patients undergoing cardiac surgery has yet to be definitively established.

Accordingly, this study aims to comprehensively evaluate the association between tramadol use and short-term outcomes, including 28-day mortality, intensive care unit (ICU) mortality, and ICU length of stay, in critically ill patients undergoing cardiac surgery. Furthermore, we compared the predictive performance of tramadol with that of other commonly used opioids, with the goal of informing more personalized and effective analgesic strategies for this vulnerable population.

## 2 Methods

### 2.1 Data source and study participants

Medical Information Mart for Intensive Care IV (MIMIC-IV) is an open-access clinical database developed by the Laboratory for Computational Physiology at the Massachusetts Institute of Technology (MIT) in collaboration with Beth Israel Deaconess Medical Center (BIDMC). It contains de-identified health data from over 40,000 ICU patients treated at BIDMC between 2008 and 2019, encompassing multidimensional information including demographics, vital signs, laboratory results, medication records, imaging reports, and hospital outcomes. As all personally identifiable information has been removed from the dataset, the need for individual informed consent was waived for this study in accordance with ethical guidelines for secondary use of de-identified data. This study was conducted in accordance with the Strengthening the Reporting of Observational Studies in Epidemiology (STROBE) guidelines and adheres to the ethical principles set forth in the Declaration of Helsinki.

Patients admitted to the ICU following cardiac surgery were included in this study based on International Classification of Diseases, Ninth and 10th Revisions (ICD-9 and ICD-10) codes (n = 4,035). Exclusion criteria comprised age <18 years, non-admission to the ICU (n = 191), ICU length of stay ≤24 h (n = 299), and implausible survival times (n = 1). After applying these criteria, a total of 3,544 patients were included in the final analysis.

### 2.2 Exposure and clinical outcome

The primary exposure of interest was postoperative tramadol use after ICU admission. To ensure appropriate temporal sequencing, medication administration records were systematically reviewed to confirm that all tramadol administrations occurred after ICU admission following cardiac surgery. Patients who received at least one dose of tramadol after ICU admission following cardiac surgery were accordingly assigned to the tramadol group (n = 1,576), whereas those who did not receive any tramadol were classified into the non-tramadol group (n = 1,968). We further characterized the exposure by calculating the cumulative dose and duration of therapy based on the medication administration records.

The primary outcome was 28-day mortality, with the observation period starting at the time of ICU admission and ending 28 days thereafter. Secondary outcomes included ICU length of stay and in-ICU mortality.

### 2.3 Data collection

We also collected demographic characteristics such as age, gender, race; insurance, marital status, smoking, drinking, and body mass index (BMI); vital signs including heart rate, respiratory rate, and mean blood pressure (MBP), and oxygen saturation (SpO_2_); comorbidities including anxiety, depression, delirium, and anemia; clinical scores including Charlson comorbidity index (CCI), Glasgow Coma Scale (GCS), sequential organ failure assessment (SOFA), Simplified Acute Physiology Score II (SAPSII), systemic inflammatory response syndrome (SIRS), and oxford acute severity of illness score (OASIS); laboratory parameters including partial pressure of oxygen (pO_2_), anion gap (AG), bicarbonate, blood urea nitrogen (BUN), phosphate, pH, glucose, prothrombin time (PT), calcium, potassium, creatinine, international normalized ratio (INR), partial pressure of carbon dioxide (pCO_2_), platelets, partial thromboplastin time (PTT), red blood cell (RBC), red cell distribution width (RDW), sodium, total bilirubin, white blood cell (WBC), and lactate; intervention including heparin, vasopressor, antibiotic, renal replacement therapy (RRT), and ventilation. As this is a retrospective study based on electronic health records, specific details regarding the assay kits or their manufacturers are not available. However, all measurements were conducted at the clinical laboratory of BIDMC, in accordance with standard clinical operating procedures and quality control protocols in place during patient care (Johnson et al., 2023).

### 2.4 Statistical analysis

Continuous data are presented as mean ± standard deviation if normality was confirmed by the Shapiro-Wilk test; otherwise, they are reported as median (interquartile range). Categorical variables are expressed as frequency (percentage). Group comparisons were conducted according to the nature of the data: independent samples t-test for normally distributed continuous variables, Mann-Whitney U test for non-normally distributed continuous variables, and chi-square or Fisher’s exact test for categorical variables, as appropriate. All statistical analyses were performed using R software (version 4.2.1) (Team, 2024), and a *P*-value <0.05 was considered statistically significant.

To evaluate the impact of tramadol use on 28-day mortality among cardiac surgery patients in the ICU, Kaplan-Meier (KM) survival curves and the log-rank test were utilized. Prior to multivariate regression analysis, variables showing significant differences in baseline analysis were selected as covariates for adjustment, and variance inflation factors (VIFs) were computed to assess potential multicollinearity among predictors (Yang et al., 2025). The proportional hazards assumption for Cox regression was examined using Schoenfeld residuals (Chen et al., 2024). Subsequently, the association between tramadol use and survival outcomes was analyzed using Cox proportional hazards models. Six adjusted models were constructed in the primary analysis to account for confounding: Model 1 adjusted for demographic characteristics, including age, sex, and insurance; Model 2 adjusted for comorbidity-related factors, including CCI, delirium, and anemia; Model 3 controlled for intervention-related variables, including ventilation, surgery type, vasopressor, and antibiotic; Model 4 included disease severity scores such as SOFA, SAPSII, SIRS, and OASIS; Model 5 incorporated laboratory indicators, including SpO_2_, bicarbonate, and total bilirubin; and Model 6 adjusted for all aforementioned covariates. Results are presented as hazard ratios (HR) with corresponding 95% confidence intervals (95%CI). Linear regression was employed to examine the association between tramadol use and length of ICU stay.

Propensity score matching (PSM) was performed to mitigate confounding by indication (Kruger et al., 2025). Propensity scores were estimated using logistic regression incorporating all aforementioned covariates. Nearest-neighbor matching with a caliper width of 0.2 was applied. Covariate balance after matching was assessed using absolute standardized differences (ASD), where an ASD <0.1 indicates adequate balance between groups (Arribas Lopez et al., 2025). To further evaluate the robustness and consistency of the observed associations, a series of sensitivity analyses were conducted, including doubly robust estimation and inverse probability weighting (IPW) based on propensity scores, as well as E-value calculations to assess the potential influence of unmeasured confounding (VanderWeele and Ding, 2017):

$$E‐value=\sqrt{\text{HR}\times \left(\text{HR}‐1\right)}$$

The following subgroups and interaction effects were assessed: age (<65 vs. ≥65), sex (male vs. female), CCI (<5 vs. ≥5), SOFA score (<3 vs. ≥3), and surgery type (coronary artery bypass grafting [CABG] vs. valve surgery).

Finally, the predictive performance of different opioids for 28-day mortality was compared using receiver operating characteristic (ROC) curves and the DeLong test. The prediction model was visualized using SHAP (SHapley Additive exPlanations) values based on logistic regression to quantify the contribution of each feature to individual predictions (Cheng et al., 2024). For variables with missing data (<20%), multiple imputation was performed using the “mice” package in R (van Buuren and Groothuis-Oudshoorn, 2011), while variables with more than 20% missing data were not included in this study.

## 3 Results

### 3.1 Characteristics of patients

**FIGURE 1:**
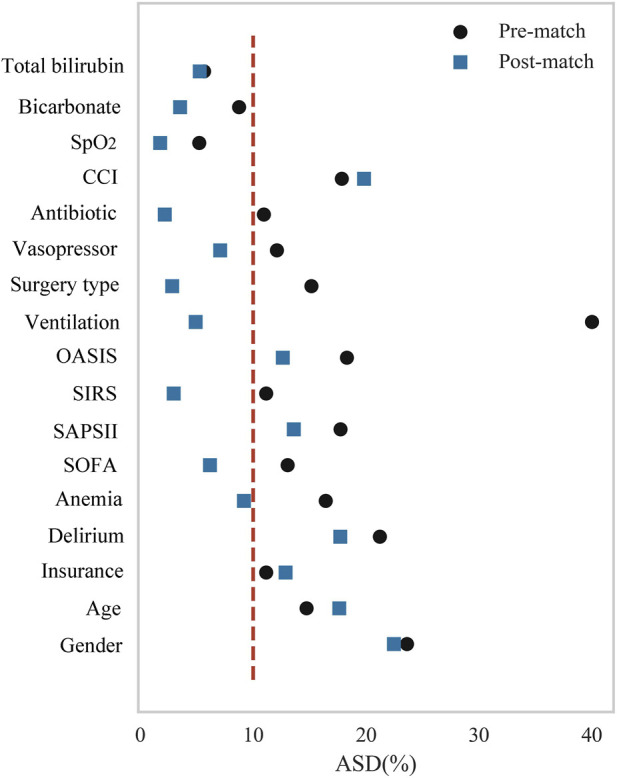
Covariate balance before and after PSM. Abbreviations: PSM, propensity score matching; ASD, absolute standardized difference; CCI, charlson comorbidity index; SOFA, sequential organ failure assessment; SAPSII, simplified acute physiology score II; SIRS, systemic inflammatory response syndrome; OASIS, oxford acute severity of illness score; SpO_2_, oxygen saturation.

Table 1 demonstrates baseline characteristics between the non-tramadol and tramadol groups. Among the 3,544 participants, 1,576 patients received tramadol. The median duration of tramadol therapy was 3.6 days (IQR: 2.1–5.3), and the median cumulative dose was 50 mg (IQR: 50–75). The violin plots regarding tramadol dose (Supplementary Figure S1A) and duration (Supplementary Figure S1B) are provided in Supplementary Figure S1. The tramadol group was older and had a higher proportion of female patients. Patients receiving tramadol were more likely to have Medicare or Medicaid insurance coverage and to undergo CABG rather than valve surgery. This group exhibited greater illness severity, as indicated by significantly higher SOFA, SAPSII, SIRS, and OASIS scores. They also had higher CCI and higher prevalences of comorbidities, including delirium and anemia. With regard to interventions, the tramadol group received vasopressors, antibiotics, and ventilation more frequently. Laboratory analyses revealed that the tramadol group had higher oxygen saturation (SpO_2_), but lower bicarbonate and total bilirubin levels. In contrast, no significant differences were observed between the groups in terms of race, marital status, smoking or drinking history, BMI, vital signs (except SpO_2_), anxiety, depression, GCS score, or multiple laboratory parameters, including blood gas measurements, electrolyte levels, renal function tests, and coagulation markers (all *P* < 0.05). These substantial baseline imbalances highlight the necessity of PSM. Accordingly, the subsequent PSM adjusted for all variables that exhibited significant differences between groups. After PSM, the two groups achieved a good balance in the vast majority of these baseline characteristics (ASD <0.1), except for age, gender, insurance, CCI, OASIS, SAPSII, and delirium, as shown in Figure 1. The specific ASD values are presented in Supplementary Table S1.

**TABLE 1:** Baseline characteristics of cardiac surgery patient cohorts before and after propensity score matching.

Variables	Before PSM			After PSM		
	Non-tramadol (n = 1968)	Tramadol (n = 1,576)	*P*	Non-tramadol (n = 1,576)	Tramadol (n = 1,576)	*P*
Age, years	67.000 [59.000,75.000]	69.000 [61.000,76.000]	<0.001	67.000 [59.000,74.000]	69.000 [61.000,76.000]	<0.001
Gender, n (%)	​	​	<0.001	​	​	<0.001
Male	1,477 (75.051)	1,013 (64.277)	​	1,175 (74.556)	1,013 (64.277)	​
Female	491 (24.949)	563 (35.723)	​	401 (25.444)	563 (35.723)	​
Race, n (%)	​	​	0.882	​	​	0.348
White	1,484 (75.407)	1,185 (75.190)	​	1,162 (73.731)	1,185 (75.190)	​
Other	484 (24.593)	391 (24.810)	​	414 (26.269)	391 (24.810)	​
Insurance, n (%)	​	​	<0.001	​	​	<0.001
Medicare/Medicaid	892 (45.325)	802 (50.888)	​	701 (44.480)	802 (50.888)	​
Other	1,076 (54.675)	774 (49.112)	​	875 (55.520)	774 (49.112)	​
Marital status, n (%)	​	​	0.972	​	​	0.745
Married	1,145 (58.181)	916 (58.122)	​	925 (58.693)	916 (58.122)	​
Other	823 (41.819)	660 (41.878)	​	651 (41.307)	660 (41.878)	​
Smoking, n (%)	​	​	0.142	​	​	0.21
No	1910 (97.053)	1,542 (97.843)	​	1,531 (97.145)	1,542 (97.843)	​
Yes	58 (2.947)	34 (2.157)	​	45 (2.855)	34 (2.157)	​
Drinking, n (%)	​	​	0.34	​	​	0.206
No	1892 (96.138)	1,505 (95.495)	​	1,519 (96.383)	1,505 (95.495)	​
Yes	76 (3.862)	71 (4.505)	​	57 (3.617)	71 (4.505)	​
BMI, kg/m^2^	28.900 [26.000,32.200]	28.750 [25.800,31.900]	0.413	29.000 [26.120,32.300]	28.750 [25.800,31.900]	0.195
Surgery type, n (%)	​	​	<0.001	​	​	0.429
CABG	978 (49.695)	902 (57.234)	​	880 (55.838)	902 (57.234)	​
Valve	990 (50.305)	674 (42.766)	​	696 (44.162)	674 (42.766)	​
Vital signs						
Heart rate, bpm	80.000 [73.000,85.000]	80.000 [73.000,85.000]	0.119	80.000 [73.000,85.000]	80.000 [73.000,85.000]	0.19
Respiratory rate, insp/min	16.000 [14.000,18.000]	16.000 [14.000,18.000]	0.481	16.000 [14.000,18.000]	16.000 [14.000,18.000]	0.934
MBP, mmHg	77.000 [70.000,85.000]	77.000 [69.333,84.667]	0.346	76.333 [69.333,84.333]	77.000 [69.333,84.667]	0.548
SpO_2_, %	100.000 [98.000,100.000]	100.000 [99.000,100.000]	0.006	100.000 [99.000,100.000]	100.000 [99.000,100.000]	0.803
Comorbidities						
Anxiety, yes, n (%)	288 (14.634)	220 (13.959)	0.569	233 (14.784)	220 (13.959)	0.509
Depression, yes, n (%)	231 (11.738)	213 (13.515)	0.112	171 (10.850)	213 (13.515)	0.022
Delirium, yes, n (%)	93 (4.726)	162 (10.279)	<0.001	87 (5.520)	162 (10.279)	<0.001
Anemia, yes, n (%)	1,274 (64.736)	1,140 (72.335)	<0.001	1,074 (68.147)	1,140 (72.335)	0.01
Clinical scores						
CCI	5.000 [4.000,7.000]	5.000 [4.000,7.000]	<0.001	5.000 [4.000,6.000]	5.000 [4.000,7.000]	<0.001
GCS	15.000 [15.000,15.000]	15.000 [15.000,15.000]	0.954	15.000 [15.000,15.000]	15.000 [15.000,15.000]	0.621
SOFA	2.000 [1.000,4.000]	3.000 [1.000,4.000]	<0.001	3.000 [1.000,4.000]	3.000 [1.000,4.000]	0.131
SAPSII	34.000 [29.000,40.000]	36.000 [30.000,43.000]	<0.001	35.000 [29.000,41.000]	36.000 [30.000,43.000]	<0.001
SIRS	3.000 [2.000,3.000]	3.000 [2.000,3.000]	0.002	3.000 [2.000,3.000]	3.000 [2.000,3.000]	0.444
OASIS	30.000 [25.000,35.000]	31.000 [26.000,37.000]	<0.001	30.000 [26.000,35.000]	31.000 [26.000,37.000]	<0.001
Laboratory tests						
pO_2_, mmHg	347.000 [263.000,404.000]	351.000 [267.000,413.000]	0.178	353.000 [266.000,408.000]	351.000 [267.000,413.000]	0.906
AG, mEq/L	13.000 [11.000,16.000]	13.000 [11.000,15.000]	0.077	13.000 [11.000,16.000]	13.000 [11.000,15.000]	0.423
Bicarbonate, mEq/L	24.000 [22.000,26.000]	24.000 [22.000,25.000]	0.006	24.000 [22.000,25.000]	24.000 [22.000,25.000]	0.197
BUN, mg/dL	17.000 [14.000,23.000]	17.000 [14.000,23.000]	0.434	17.000 [13.000,22.000]	17.000 [14.000,23.000]	0.032
Phosphate, mg/dL	3.500 [3.100,4.000]	3.500 [3.100,4.000]	0.484	3.500 [3.100,4.000]	3.500 [3.100,4.000]	0.695
pH	7.400 [7.370,7.430]	7.400 [7.370,7.430]	0.531	7.400 [7.370,7.430]	7.400 [7.370,7.430]	0.513
Glucose, mg/dL	111.000 [97.000,144.000]	113.000 [98.000,146.000]	0.522	112.000 [97.000,147.000]	113.000 [98.000,146.000]	0.915
PT, sec	15.200 [13.900,16.800]	15.400 [13.900,17.100]	0.171	15.400 [13.900,16.900]	15.400 [13.900,17.100]	0.871
Calcium, mg/dL	8.530 [8.150,9.000]	8.500 [8.100,9.000]	0.203	8.500 [8.100,9.000]	8.500 [8.100,9.000]	0.942
Potassium, mEq/L	4.200 [4.000,4.500]	4.200 [4.000,4.600]	0.524	4.200 [4.000,4.500]	4.200 [4.000,4.600]	0.905
Creatinine, mg/dL	0.900 [0.800,1.200]	0.900 [0.800,1.200]	0.879	0.900 [0.800,1.100]	0.900 [0.800,1.200]	0.236
INR	1.200 [1.100,1.400]	1.200 [1.100,1.400]	0.38	1.200 [1.100,1.400]	1.200 [1.100,1.400]	0.141
pCO_2_, mmHg	40.900 [37.000,44.000]	40.000 [37.000,44.000]	0.204	40.000 [37.000,44.000]	40.000 [37.000,44.000]	0.556
Platelets, K/μL	171.000 [132.000,215.000]	171.000 [131.000,217.000]	0.757	171.000 [133.000,215.000]	171.000 [131.000,217.000]	0.679
PTT, sec	13.300 [11.900,15.500]	13.200 [11.900,15.400]	0.428	13.400 [11.900,15.500]	13.200 [11.900,15.400]	0.143
RBC, m/μL	3.720 [3.090,4.330]	3.670 [3.070,4.310]	0.191	3.710 [3.070,4.340]	3.670 [3.070,4.310]	0.315
RDW, %	13.400 [12.700,14.500]	13.400 [12.800,14.500]	0.293	13.300 [12.700,14.300]	13.400 [12.800,14.500]	0.002
Sodium, mEq/L	139.000 [137.000,141.000]	139.000 [137.000,141.000]	0.085	139.000 [137.000,141.000]	139.000 [137.000,141.000]	0.129
Total bilirubin, mg/dL	0.600 [0.430,0.770]	0.590 [0.400,0.730]	0.017	0.600 [0.430,0.770]	0.590 [0.400,0.730]	0.049
WBC, K/μL	8.900 [6.800,12.700]	9.000 [6.900,12.400]	0.715	9.000 [7.000,13.200]	9.000 [6.900,12.400]	0.276
Lactate, mmol/L	1.400 [1.100,1.870]	1.400 [1.100,1.800]	0.58	1.400 [1.100,1.900]	1.400 [1.100,1.800]	0.727
Intervention						
Heparin, yes, n (%)	1,426 (72.459)	1,181 (74.937)	0.097	1,123 (71.256)	1,181 (74.937)	0.02
Vasopressor, yes, n (%)	386 (19.614)	388 (24.619)	<0.001	341 (21.637)	388 (24.619)	0.047
Antibiotic, yes, n (%)	1720 (87.398)	1,431 (90.799)	<0.001	1,421 (90.165)	1,431 (90.799)	0.544
RRT, yes, n (%)	26 (1.321)	27 (1.713)	0.339	21 (1.332)	27 (1.713)	0.383
Ventilation, yes, n (%)	1728 (87.805)	1,543 (97.906)	<0.001	1,531 (97.145)	1,543 (97.906)	0.169
Outcomes						
28-day mortality, yes, n (%)	47 (2.388)	15 (0.952)	0.001	35 (2.221)	15 (0.952)	0.004
ICU mortality, yes, n (%)	13 (0.661)	8 (0.508)	0.555	12 (0.761)	8 (0.508)	0.37
Length of ICU stay, days	1.773 [1.257,2.987]	2.092 [1.301,3.448]	<0.001	1.673 [1.249,3.037]	2.092 [1.301,3.448]	<0.001

Median [IQR] for continuous variables and counts (percentage) for categorical variables. Abbreviations: IQR, interquartile range; PSM, propensity score matching; BMI, body mass index; CABG, coronary artery bypass grafting; MBP, mean blood pressure; SpO_2_, oxygen saturation; CCI, charlson comorbidity index; GCS, glasgow coma scale; SOFA, sequential organ failure assessment; SAPSII, simplified acute physiology score II; SIRS, systemic inflammatory response syndrome; OASIS, oxford acute severity of illness score; pO_2_, partial pressure of oxygen; AG, anion gap; BUN, blood urea nitrogen; PT, prothrombin time; INR, international normalized ratio; pCO_2_, partial pressure of carbon dioxide; PTT, partial thromboplastin time; RBC, red blood cell; RDW, red cell distribution width; WBC, white blood cell; RRT, renal replacement therapy; ICU, intensive care unit.

### 3.2 Primary outcome

**FIGURE 2:**
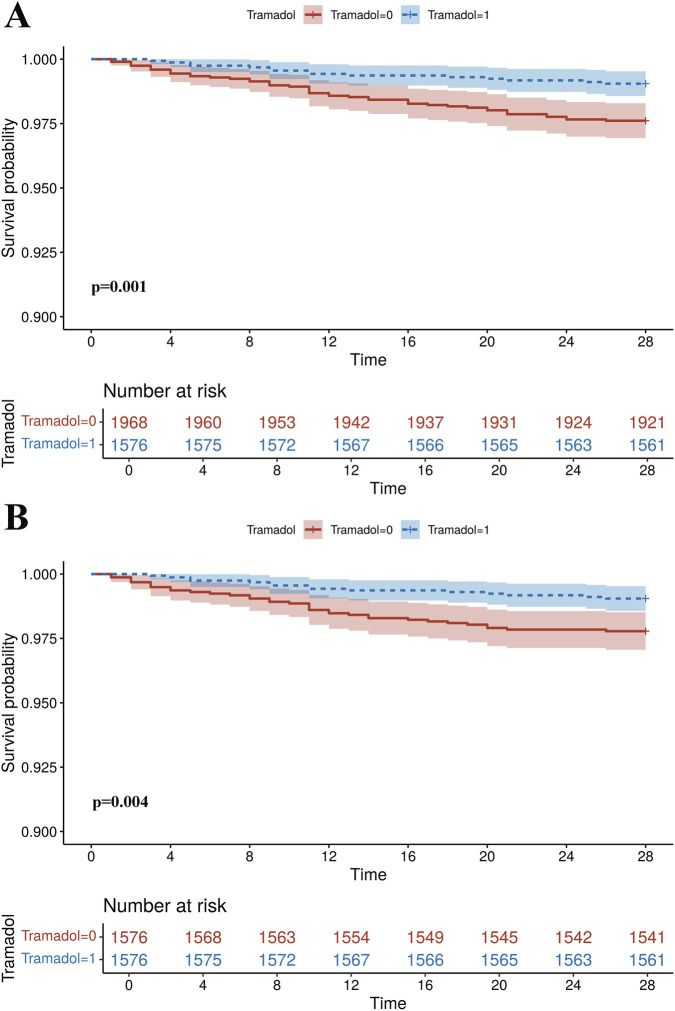
Kaplan–Meier curves of the tramadol group and non-tramadol group for 28-day mortality. **(A)** Original cohort. **(B)** PSM cohort. Abbreviations: PSM, propensity score matching.

KM curves showed that the tramadol group had a higher 28-day survival probability compared to the non-tramadol group (all *P* < 0.05), both before and after PSM (Figures 2A,B). The absence of multicollinearity is confirmed, as all VIFs for the covariates are below 5. As shown in Table 2, the association between tramadol use and reduced 28-day mortality remained consistently significant across all statistical models, both before and after PSM. Before PSM, tramadol use was associated with a significant protective effect, with HRs ranging from 0.308 to 0.407 across the six models. The greatest risk reduction was observed in Model 6—which included adjustments for all covariates—demonstrating a 69.2% (HR = 0.308, 95%CI: 0.166–0.572) decrease in mortality risk. After PSM, the protective association persisted with high consistency, with HRs ranging from 0.305 to 0.430. Notably, Model 6 continued to yield the strongest effect post-matching, with an HR of 0.305 (95%CI: 0.161–0.577). The robustness of this association across diverse adjustment strategies, including demographic characteristics, comorbidities, interventions, disease severity scores, and laboratory parameters, supports a consistent and substantial protective effect of tramadol use against 28-day mortality among patients undergoing cardiac surgery.

**TABLE 2:** Association between tramadol use and 28-day mortality among ICU patients undergoing cardiac surgery.

Models	Before PSM		After PSM	
	HR (95%CI)	*P*-value	HR (95%CI)	*P*-value
Model 1	0.364 (0.203, 0.652)	<0.001	0.366 (0.199, 0.672)	0.001
Model 2	0.337 (0.188, 0.606)	<0.001	0.361 (0.196, 0.663)	0.001
Model 3	0.387 (0.214, 0.700)	<0.001	0.382 (0.208, 0.700)	0.002
Model 4	0.317 (0.176, 0.571)	0.002	0.353 (0.192, 0.649)	<0.001
Model 5	0.407 (0.227, 0.729)	<0.001	0.430 (0.235, 0.789)	0.006
Model 6	0.308 (0.166, 0.572)	<0.001	0.305 (0.161, 0.577)	<0.001

Model 1 was adjusted for demographic characteristics, including age, gender, and insurance; Model 2 was adjusted for comorbidities-related factors, including CCI, delirium, and anemia; Model 3 was adjusted for intervention-related factors include ventilation, surgery type, vasopressor, and antibiotic; Model 4 was adjusted for other disease severity scores, including SOFA, SAPSII, SIRS, and OASIS; Model 5 was adjusted for laboratory indicators, including SpO_2_, bicarbonate, total bilirubin; Model 6 was adjusted for all covariates. Abbreviations: HR, hazard ratio; CI, confidence interval; ICU, intensive care unit; PSM, propensity score matching; CCI, charlson comorbidity index; SOFA, sequential organ failure assessment; SAPSII, simplified acute physiology score II; SIRS, systemic inflammatory response syndrome; OASIS, oxford acute severity of illness score; SpO_2_, oxygen saturation.

**FIGURE 3:**
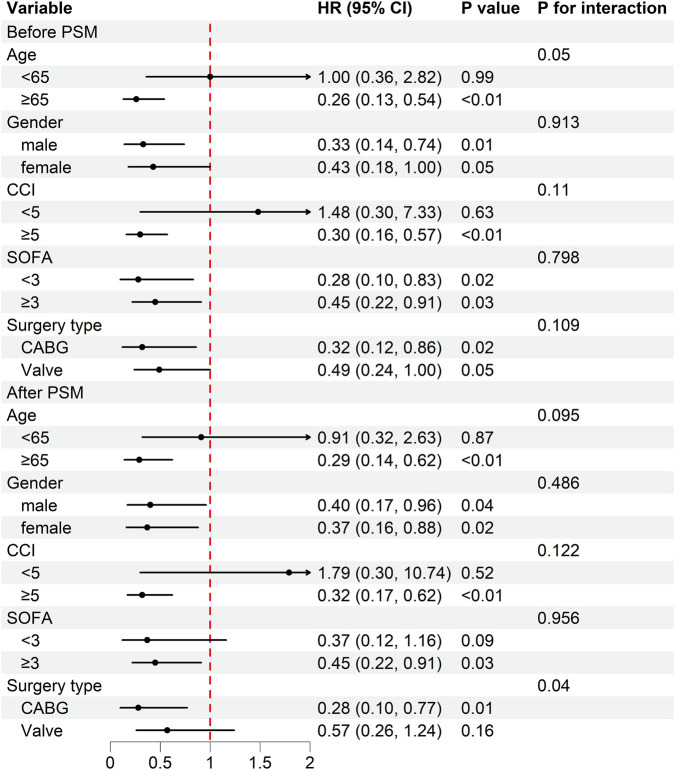
Subgroup analysis of the association between tramadol and 28-day mortality before and after PSM. Reference group is non-tramadol group. Adjusted for all covariates. Abbreviations: HR, hazard ratio; CI, confidence interval; PSM, propensity score matching; CCI, charlson comorbidity index; SOFA, sequential organ failure assessment; CABG, coronary artery bypass grafting.

We subsequently conducted subgroup analyses of 28-day mortality in cardiac surgery patients according to age, gender, SOFA scores, CCI scores, and type of surgery (Figure 3). Prior to PSM, tramadol demonstrated a significant protective effect (HR < 1) across most subgroups, with particularly strong associations observed in patients aged ≥65 years (HR = 0.26, 95%CI: 0.13–0.54), males (HR = 0.33, 95%CI: 0.14–0.74), those with CCI ≥5 (HR = 0.30, 95%CI: 0.16–0.57), both SOFA groups (SOFA <3: HR = 0.28, 95%CI: 0.10–0.83; SOFA ≥3: HR = 0.45, 95%CI: 0.22–0.91), and patients undergoing CABG surgery (HR = 0.32, 95%CI: 0.12–0.86). After PSM, the protective association remained consistent in most subgroups, with significant risk reductions maintained in patients aged ≥65 years (HR: 0.29, 95%CI: 0.14–0.62), both genders (males: HR = 0.40, 95%CI: 0.17–0.96; females: HR = 0.37, 95%CI: 0.16–0.88), those with CCI ≥5 (HR = 0.32, 95%CI: 0.17–0.62), those with SOFA ≥3 (HR = 0.45, 95%CI: 0.22–0.91), and CABG patients (HR = 0.28, 95%CI: 0.10–0.77). Notably, a significant interaction was observed for surgery type after PSM (*P* for interaction = 0.04), suggesting differential effects between CABG and valve surgery patients. The consistency of the protective association across most subgroups reinforces the robustness of the primary findings.

### 3.3 Secondary outcomes

Table 3 summarizes the results of the secondary outcomes. For ICU mortality, tramadol use showed no significant association either before or after PSM, with HRs of 0.420 (95%CI: 0.140–1.296, *P* = 0.133) before matching and 0.410 (95%CI: 0.134–1.253, *P* = 0.118) after matching. Similarly, for length of ICU stay, tramadol use demonstrated no significant effect, with estimates of 0.059 (95%CI: −0.232 to 0.350, *P* = 0.689) before matching and 0.065 (95%CI: −0.234 to 0.363, *P* = 0.670) after matching. These results indicate that tramadol use was not significantly associated with either ICU mortality or the duration of ICU stay in cardiac surgery patients, and this lack of association remained consistent after PSM adjustment.

**TABLE 3:** Association between tramadol use and secondary outcomes among cardiac surgery patients.

Secondary outcomes	Before PSM		After PSM	
	Estimate (95%CI)	*P*-value	Estimate (95%CI)	*P*-value
ICU mortality	0.420 (0.140, 1.296)	0.133	0.410 (0.134, 1.253)	0.118
Length of ICU stay	0.059 (−0.232, 0.350)	0.689	0.065 (−0.234, 0.363)	0.670

Adjusted for age, gender, insurance, CCI, delirium, anemia, ventilation, surgery type, vasopressor, antibiotic; SOFA, SAPSII, SIRS, OASIS, SpO_2_, bicarbonate, and total bilirubin. Abbreviations: CI, confidence interval; ICU, intensive care unit; PSM, propensity score matching; CCI, charlson comorbidity index; SOFA, sequential organ failure assessment; SAPSII, simplified acute physiology score II; SIRS, systemic inflammatory response syndrome; OASIS, oxford acute severity of illness score; SpO_2_, oxygen saturation.

### 3.4 Sensitivity analysis

In the primary analysis, we examined the association between tramadol use and short-term outcomes among patients undergoing cardiac surgery using both conventional multivariate regression and PSM-adjusted regression. To assess the robustness of our findings, we further employed three propensity score–based analytical approaches: IPW, doubly robust estimation with unbalanced covariates, and doubly robust estimation with all covariates, along with E-value calculations to evaluate the potential influence of unmeasured confounding. Tramadol use was significantly associated with a reduced risk of 28-day mortality but showed no significant association with ICU mortality or length of ICU stay. These results were consistently replicated in IPW and doubly robust analyses (Table 4). Specifically, for the primary outcome of 28-day mortality, IPW yielded a HR of 0.317 (95% CI: 0.174–0.576, *P* < 0.001). Doubly robust estimation methods—both adjusting for unbalanced covariates (HR = 0.340, 95% CI: 0.189–0.612) and for all covariates (HR = 0.341, 95% CI: 0.195–0.598)—produced comparable results, with both models reaching statistical significance (*P* < 0.001). The E-values for the three outcomes were 5.948 (28-day mortality), 4.115 (ICU mortality), and 4.350 (length of ICU stay), respectively. Such high E-values suggest that any unmeasured confounder would need to exhibit a strong association with both tramadol use and the outcome to fully account for the observed protective effect, further confirming that the impact of unknown confounding on the results is small.

**TABLE 4:** Comparative analysis of the association between tramadol use and outcomes using different methods.

Outcomes	Estimate	Lower 95%CI	Upper 95%CI	*P*-value
28-day mortality				
Multivariate	0.308	0.166	0.572	<0.001
Propensity score matching	0.305	0.161	0.577	<0.001
Propensity score IPW	0.317	0.174	0.576	<0.001
Doubly robust with unbalanced covariates	0.340	0.189	0.612	<0.001
Doubly robust with all covariates	0.341	0.195	0.598	<0.001
E-value	5.948	​	2.892	​
ICU mortality				
Multivariate	0.420	0.140	1.296	0.133
Propensity score matching	0.410	0.134	1.253	0.118
Propensity score IPW	0.308	0.099	0.957	0.042
Doubly robust with unbalanced covariates	0.483	0.199	1.173	0.108
Doubly robust with all covariates	0.497	0.227	1.089	0.081
E-value	4.115	​	1.000	​
Length of ICU stay				
Multivariate	0.059	−0.232	0.350	0.689
Propensity score matching	0.065	−0.234	0.363	0.670
Propensity score IPW	0.041	0.138	0.295	0.768
Doubly robust with unbalanced covariates	0.067	−0.229	0.363	0.656
Doubly robust with all covariates	0.055	−0.194	0.304	0.664
E-value	4.350	​	1.000	​

Adjusted for age, gender, insurance, CCI, delirium, anemia, ventilation, surgery type, vasopressor, antibiotic; SOFA, SAPSII, SIRS, OASIS, SpO_2_, bicarbonate, and total bilirubin. Abbreviations: CI, confidence interval; ICU, intensive care unit; IPW, inverse probability weighting; CCI, charlson comorbidity index; SOFA, sequential organ failure assessment; SAPSII, simplified acute physiology score II; SIRS, systemic inflammatory response syndrome; OASIS, oxford acute severity of illness score; SpO_2_, oxygen saturation.

### 3.5 Comparison of the predictive performance of tramadol and other opioids in predicting 28-day mortality and model explanation

**FIGURE 4:**
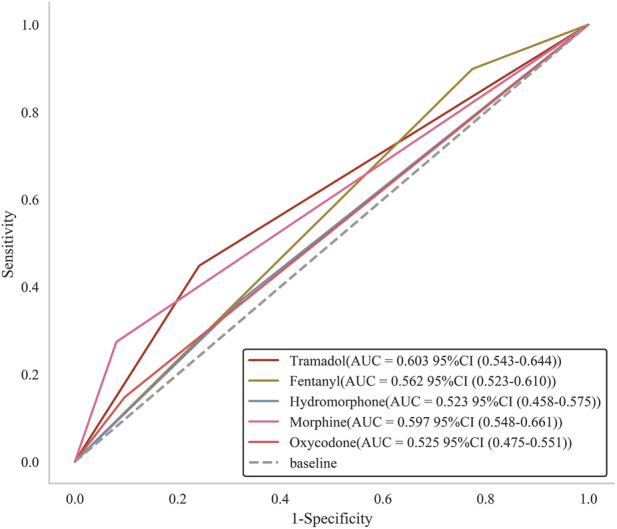
The comparative performance of various opioids in predicting 28-day mortality among ICU patients undergoing cardiac surgery. Abbreviations: ICU, intensive care unit; AUC, area under the curve.

To evaluate whether tramadol exhibits a distinct predictive advantage for 28-day mortality compared to other opioid analgesics, we performed a comparative analysis using ROC curve analysis in conjunction with the DeLong test. Tramadol achieved the highest area under the curve (AUC) of 0.603 (95% CI: 0.543–0.644) among all opioids, indicating better discriminative ability for mortality prediction (Figure 4). While morphine showed higher specificity (0.919 vs. 0.758) and fentanyl demonstrated higher sensitivity (0.899 vs. 0.448), tramadol exhibited the highest Youden index of 0.206, which combines sensitivity and specificity into a single measure of overall diagnostic effectiveness (Table 5). The Delong test results further support tramadol’s superior predictive performance, showing statistically significant differences compared to hydromorphone (*P* = 0.001) and oxycodone (*P* = 0.017). Although tramadol’s AUC was not significantly different from fentanyl (*P* = 0.232) and morphine (*P* = 0.061) based on conventional significance thresholds, it nevertheless achieved the highest point estimate for AUC among all opioids evaluated.

**TABLE 5:** Performance of opioids for predicting 28-day mortality.

Opioids	AUC (95%CI)	Sensitivity	Specificity	Youden index	Accuracy
Tramadol	0.603 (0.543–0.644)	0.448	0.758	0.206	0.454
Fentanyl	0.562 (0.523–0.610)	0.899	0.226	0.124	0.878
Hydromorphone	0.523 (0.458–0.575)	0.371	0.674	0.045	0.716
Morphine	0.597 (0.548–0.661)	0.274	0.919	0.193	0.908
Oxycodone	0.525 (0.475–0.551)	0.148	0.903	0.051	0.142

Abbreviations: CI, confidence interval; AUC, area under the curve.

**FIGURE 5:**
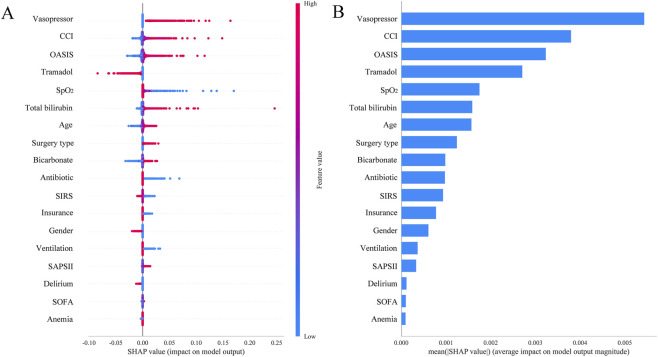
Visually interpret the predicting model using SHAP. **(A)** SHAP summary plot. **(B)** SHAP importance plot. The y-axis indicates the variables ranked by importance. The x-axis represents the SHAP value; positive values indicate increased risk of mortality, while negative values indicate decreased risk. Color reflects feature value: red for high (or “Yes”), blue for low (or “No”).

Finally, the SHAP analysis based on logistic regression was used to visualize and explain the predictive model. The SHAP feature importance plot (Figure 5B) shows that vasopressor was the most important variable in predicting 28-day mortality, followed by CCI and OASIS scores. Notably, the use of tramadol ranked fourth in the feature importance ranking, indicating its significant contribution to the model prediction. The SHAP summary plot (Figure 5A) further reveals the direction of influence of each variable on the prediction results. For tramadol, the red dots (representing the use of tramadol) are mainly concentrated in the negative region of the SHAP value, suggesting that the use of tramadol is associated with a lower risk of death and is the highest-ranked protective factor among all variables. This finding is consistent with the results of the previous multivariate regression analysis, further supporting the potential protective role of tramadol in reducing postoperative mortality risk.

## 4 Discussion

This study comprehensively evaluated the impact of tramadol on 28-day mortality in patients following cardiac surgery using various statistical methods and machine learning techniques. Multivariate Cox regression, PSM, and a series of sensitivity analyses consistently demonstrated that tramadol use was associated with a significant reduction in 28-day mortality risk. Subgroup analysis further confirmed the robustness of this association across key subgroups including age, gender, comorbidity burden, and surgery type, with a particularly pronounced protective effect observed in CABG patients. Compared to other opioids, tramadol demonstrated the best discriminative performance for predicting mortality with the highest AUC. SHAP interpretability analysis identified tramadol as the fourth most important predictive feature, with its contribution direction consistent with reduced mortality risk. Secondary outcomes analysis showed that tramadol had no significant effect on ICU mortality or length of stay.

Tramadol use may influence patient survival outcomes, although the direction of this effect—beneficial or harmful—varies across different clinical populations. A Danish cohort study leveraging electronic health records comprehensively examined the associations between various opioid therapies and 90-day mortality and hospital readmission rates (Winther et al., 2024), revealing that, compared with morphine, tramadol was associated with a higher risk of readmission but a significantly lower risk of 90-day mortality. Notably, in patients with rheumatoid arthritis and osteoarthritis, tramadol use has been linked to increased mortality (Gau et al., 2022; Zeng et al., 2019; Jeong et al., 2019), highlighting population-specific risks. Evidence regarding the impact of tramadol on surgical patients remains limited. To our knowledge, our study is the first to demonstrate that tramadol use is associated with improved survival outcomes in patients undergoing cardiac surgery. These findings align with those of Kim et al., who reported reduced risks of postoperative recurrence and mortality among patients receiving tramadol during breast cancer surgery (Kim et al., 2019). In contrast, our results diverge from studies involving non-thoracic surgical populations (Cha et al., 2021; Yoo et al., 2021; Muhammad et al., 2025), potentially due to differences in surgical site, underlying pathophysiology, or patient characteristics.

This study observed a significant association between tramadol use and reduced 28-day mortality, a finding potentially attributable to its distinct physiological and pharmacological properties. First, compared with traditional strong opioids such as morphine and fentanyl, tramadol is associated with minimal risk of respiratory depression (Beakley et al., 2015; Gustafsson et al., 2023). In the ICU setting, respiratory depression can precipitate pulmonary complications—including atelectasis and pneumonia—and delay extubation, both of which are linked to increased mortality. Thus, tramadol may better preserve spontaneous ventilation and reduce the incidence of these adverse outcomes. Second, unlike many other opioids, tramadol does not suppress immune function (Saeed et al., 2021); instead, it enhances natural killer (NK) cell activity, promotes lymphocyte proliferation, and increases interleukin-2 secretion (Saeed et al., 2021; Sayed et al., 2018), all of which play critical roles in immune regulation. Furthermore, evidence suggests that tramadol exerts anticoagulant and cardioprotective effects, potentially reducing thromboembolic risk in cardiac surgery patients. *In vitro* studies have demonstrated that tramadol inhibits whole-blood coagulation in a concentration-dependent manner among women with gynecological malignancies (Bilir et al., 2014). Animal models further indicate that tramadol significantly lowers lactate dehydrogenase (LDH) levels, supporting its cardioprotective potential (Sen et al., 2020; El-Ashmawy et al., 2021). Additionally, tramadol exhibits neuroprotective properties by mitigating oxidative stress damage (Sen et al., 2020; Dhull and Kumar, 2018). Its dual inhibition of serotonin and norepinephrine reuptake not only contributes to cardiovascular stability in postoperative patients but may also positively influence mood regulation (Osman and Mustafa, 2018). Collectively, tramadol offers a favorable safety profile with respect to respiratory and immune function, while providing multimodal pharmacological benefits including analgesia, anticoagulation, cardioprotection, antioxidant activity, and potential antidepressant effects.

It is noteworthy that although tramadol was associated with reduced 28-day mortality, no significant association was observed in ICU mortality or length of stay. This discrepancy likely reflects the distinct physiological challenges inherent in the acute critical phase versus the sub-acute recovery phase. ICU mortality is predominantly influenced by immediate and severe complications—such as refractory shock or hemorrhage—in which the impact of analgesic selection may be negligible compared to life-sustaining organ support interventions (Cabrera et al., 2024). In contrast, 28-day mortality encompasses outcomes during the sub-acute recovery period, often occurring after transfer from the ICU to general wards. During the sub-acute phase, tramadol’s safety profile may confer cumulative survival benefits. Its minimal respiratory depression could reduce late-onset pulmonary complications. Additionally, its lower sedative potency may promote earlier mobilization, thereby mitigating risks associated with prolonged immobility (Bosco et al., 2024). Consequently, tramadol’s protective effect appears to operate primarily through enhancing resilience to sub-acute complications—effectively preventing “failure to rescue”—rather than altering the trajectory of acute critical illness.

The subgroup analysis not only confirmed the robustness of the main effect across diverse patient populations but also revealed significant heterogeneity according to surgery type. Tramadol demonstrated a more pronounced protective effect in patients undergoing CABG, which may be attributed to a precise alignment between its pharmacological profile and the distinct pathophysiological characteristics and critical risk mitigation needs of CABG patients. Specifically, tramadol’s minimal respiratory depression, limited immunosuppression, and favorable hemodynamic stability closely align with the core objectives of perioperative management in CABG (Altun et al., 2017). In contrast, patients undergoing valve surgery require more complex and stringent hemodynamic control (Nuche et al., 2024), and current evidence suggests that tramadol’s pharmacological properties may be insufficient to meet the demands for fine-tuned physiological regulation in this population.

The findings of this study offer potential clinical implications for the selection of perioperative analgesic regimens in cardiac surgery. Our results suggest that selecting tramadol as an analgesic after cardiac surgery might represent a favorable option for balancing analgesic efficacy and safety. Its unique dual mechanism of action not only provides effective analgesia but may also indirectly contribute to improved patient outcomes by mitigating common adverse effects associated with opioids, such as respiratory depression and impaired gastrointestinal motility. Specifically, for patients at risk of respiratory complications or those requiring rapid extubation, tramadol could theoretically offer particular advantages. However, given the observational nature of this study, these results should be viewed as hypothesis-generating. Consequently, tramadol could be considered as a potential protective analgesic option within the context of personalized pain management plans, pending further validation in prospective clinical trials.

Several limitations of this study must be acknowledged when interpreting its conclusions. First, this is a retrospective observational study, and inherent biases cannot be completely eliminated. However, the high E-value (5.948) and the consistent results from the doubly robust estimation indicate that the influence of residual confounding is minimal. Second, the single-center nature of the data may limit the generalizability of our findings. Third, given the wide variety of sedatives and analgesics used in the ICU, most often in combination regimens, isolating the independent effect of any single agent remains challenging. Furthermore, although PSM was utilized to mitigate confounding by indication, the potential for selection bias remains. It is possible that tramadol was preferentially administered to patients with specific pain profiles or clinical trajectories that are not fully captured by severity scores like SOFA or OASIS. However, it is worth noting that baseline characteristics in our cohort showed that tramadol users had higher severity scores and rates of mechanical ventilation than non-users, suggesting that the drug was not exclusively reserved for ‘more stable’ patients. Nevertheless, as with all observational studies, we cannot rule out the influence of unmeasured confounders, such as the timing of extubation or the transition from intravenous to oral analgesics, which warrants cautious interpretation of the causal relationship. Finally, a principal limitation of this study lies in defining tramadol exposure as a binary variable (yes/no), without quantification of dosage, cumulative exposure, or duration. This approach precluded assessment of potential dose-response relationships and differential effects between short-term versus prolonged regimens. Future research should focus on multicenter prospective cohort studies that systematically capture data on dosage, cumulative exposure, and treatment duration to validate the robustness of these findings; randomized controlled trials comparing tramadol with other opioids on clinically meaningful endpoints; and mechanistic investigations into the biological pathways underlying its protective effects, such as through the measurement of biomarkers related to inflammation and stress response.

## 5 Conclusion

This study found that tramadol use was associated with a lower 28-day mortality rate among patients undergoing cardiac surgery, but not with ICU mortality or ICU length of stay. These findings provide clinical insights for postoperative pain management in cardiac surgery patients, suggesting that tramadol may play a potential protective role in improving outcomes for critically ill individuals.

## Data Availability

The raw data supporting the conclusions of this article will be made available by the authors, without undue reservation.

## Supplementary material

Supplementary Figure S1 Violin plots for the distribution of tramadol usage cumulative dose and duration. **(A)** Cumulative dose. **(B)** Duration.
