# Supplementary material for: Tramadol use is associated with reduced 28-day mortality in ICU patients after cardiac surgery: a retrospective study based on the MIMIC-IV database
Source: Front Pharmacol. 2026 Jan 16;17:1770570. doi: 10.3389/fphar.2026.1770570 (PMC12855536; doi:10.3389/fphar.2026.1770570)
Supplement: Supplementary file 1 [file Supplementaryfile1.docx]

**Supplementary Table 1.** Evaluation of covariate balance in the original and propensity score-matched cohorts.

|  | Before PSM | | |  | After PSM | | |
| --- | --- | --- | --- | --- | --- | --- | --- |
|  | Non-Tramadol (n=1968) | Tramadol (n=1576) | ASD |  | Non-Tramadol (n=1576) | Tramadol (n=1576) | ASD |
| Age, years | 67.000[59.000,75.000] | 69.000[61.000,76.000] | 0.147 |  | 67.000[59.000,74.000] | 69.000[61.000,76.000] | 0.176 |
| Gender, n (%) |  |  | 0.236 |  |  |  | 0.224 |
| Male | 1477(75.051) | 1013(64.277) |  |  | 1175(74.556) | 1013(64.277) |  |
| Female | 491(24.949) | 563(35.723) |  |  | 401(25.444) | 563(35.723) |  |
| Insurance, n (%) |  |  | 0.129 |  |  |  | 0.129 |
| Medicare/Medicaid | 892(45.325) | 802(50.888) |  |  | 701(44.480) | 802(50.888) |  |
| Other | 1076(54.675) | 774(49.112) |  |  | 875(55.520) | 774(49.112) |  |
| Surgery type, n (%) |  |  | 0.152 |  |  |  | 0.028 |
| CABG | 978(49.695) | 902(57.234) |  |  | 880(55.838) | 902(57.234) |  |
| Valve | 990(50.305) | 674(42.766) |  |  | 696(44.162) | 674(42.766) |  |
| SpO_2_, % | 100.000[98.000,100.000] | 100.000[99.000,100.000] | 0.052 |  | 100.000[99.000,100.000] | 100.000[99.000,100.000] | 0.017 |
| Delirium, yes, n (%) | 93(4.726) | 162(10.279) | 0.212 |  | 87(5.520) | 162(10.279) | 0.177 |
| Anemia, yes, n (%) | 1274(64.736) | 1140(72.335) | 0.164 |  | 1074(68.147) | 1140(72.335) | 0.092 |
| CCI | 5.000[4.000,7.000] | 5.000[4.000,7.000] | 0.198 |  | 5.000[4.000,6.000] | 5.000[4.000,7.000] | 0.198 |
| SOFA | 2.000[1.000,4.000] | 3.000[1.000,4.000] | 0.130 |  | 3.000[1.000,4.000] | 3.000[1.000,4.000] | 0.062 |
| SAPSII | 34.000[29.000,40.000] | 36.000[30.000,43.000] | 0.177 |  | 35.000[29.000,41.000] | 36.000[30.000,43.000] | 0.136 |
| SIRS | 3.000[2.000,3.000] | 3.000[2.000,3.000] | 0.111 |  | 3.000[2.000,3.000] | 3.000[2.000,3.000] | 0.030 |
| OASIS | 30.000[25.000,35.000] | 31.000[26.000,37.000] | 0.183 |  | 30.000[26.000,35.000] | 31.000[26.000,37.000] | 0.126 |
| Bicarbonate, mEq/L | 24.000[22.000,26.000] | 24.000[22.000,25.000] | 0.087 |  | 24.000[22.000,25.000] | 24.000[22.000,25.000] | 0.035 |
| Total bilirubin, mg/dL | 0.600[0.430,0.770] | 0.590[0.400,0.730] | 0.056 |  | 0.600[0.430,0.770] | 0.590[0.400,0.730] | 0.052 |
| Vasopressor, yes, n (%) | 386(19.614) | 388(24.619) | 0.121 |  | 341(21.637) | 388(24.619) | 0.071 |
| Antibiotic, yes, n (%) | 1720(87.398) | 1431(90.799) | 0.109 |  | 1421(90.165) | 1431(90.799) | 0.022 |
| Ventilation, yes, n (%) | 1728(87.805) | 1543(97.906) | 0.400 |  | 1531(97.145) | 1543(97.906) | 0.049 |

Median [IQR] for continuous variables and counts (percentage) for categorical variables. Abbreviations: ASD, absolute standardized differences; IQR, interquartile range; PSM, propensity score matching; CABG, coronary artery bypass grafting; SpO_2_, oxygen saturation; CCI, charlson comorbidity index; SOFA, sequential organ failure assessment; SAPSII, simplified acute physiology score II; SIRS, systemic inflammatory response syndrome; OASIS, oxford acute severity of illness score.
