# Supplementary figures and images for: Tramadol use is associated with reduced 28-day mortality in ICU patients after cardiac surgery: a retrospective study based on the MIMIC-IV database
Source: Front Pharmacol. 2026 Jan 16;17:1770570. doi: 10.3389/fphar.2026.1770570 (PMC12855536; doi:10.3389/fphar.2026.1770570)

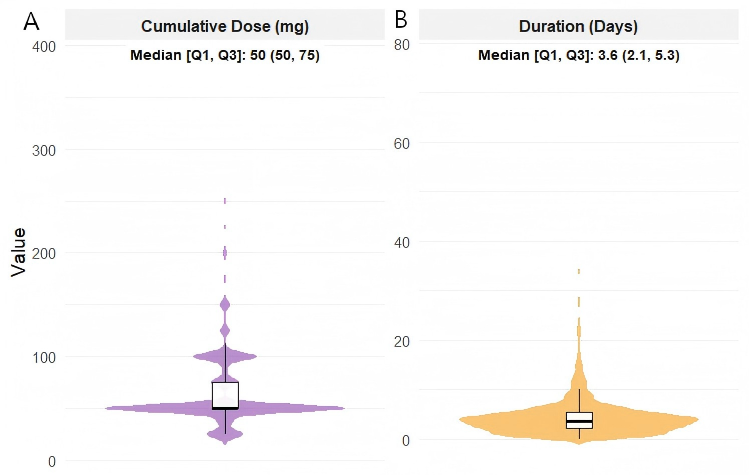

Supplement: Supplementary file 2 [file Image1.tif]
